# Ten years of specialized adult care for phenylketonuria – a single-centre experience

**DOI:** 10.1186/s13023-016-0410-6

**Published:** 2016-03-24

**Authors:** Ulrike Mütze, Alena Gerlinde Thiele, Christoph Baerwald, Uta Ceglarek, Wieland Kiess, Skadi Beblo

**Affiliations:** Department of Women and Child Health, Hospital for Children and Adolescents, Centre for Paediatric Research Leipzig (CPL), University Hospitals, University of Leipzig, Liebigstr. 20 a, 04103 Leipzig, Germany; Department of Internal Medicine, University Hospitals, University of Leipzig, Liebigstr.20, 04103 Leipzig, Germany; Institute of Laboratory Medicine, Clinical Chemistry and Molecular Diagnostics, University Hospitals, University of Leipzig, Paul-List-Str. 13-15, 04103 Leipzig, Germany; Division of Neuropediatrics and Inherited Metabolic Diseases, Department of General Pediatrics, University Children’s Hospital Heidelberg, Im Neuenheimer Feld 430, 69120 Heidelberg, Germany

**Keywords:** Phenylketonuria, Adult care, Transition, Metabolic control, Maternal PKU syndrome, Sociodemographic outcome

## Abstract

**Background:**

Specialized adult care of phenylketonuria (PKU) patients is of increasing importance. Adult outpatient clinics for inherited errors of metabolism can help to achieve this task, but experience is limited. Ten years after establishment of a coordinated transition process and specialised adult care for inherited metabolic diseases, adult PKU care was evaluated with respect to metabolic control, therapy satisfaction, life satisfaction, sociodemographic data, economical welfare as well as pregnancy outcome.

**Methods:**

All PKU patients transferred from paediatric to adult care between 2005 and 2015 were identified. A retrospective data analysis and a cross-sectional survey in a sub-cohort of 30 patients including a questionnaire for assessing quality of life (FLZm) were performed as a single-centre investigation at the metabolic department of the University Hospital Leipzig, Germany. For statistical analysis, Mann-Whitney-*U*-test, *t*-test for independent samples, ANOVA and chi square test were used as appropriate.

**Results:**

96 PKU patients (56 females/40 males; median age 32 years, range 18–62) were included. In the last 3-year period, 81 % of the transferred patients still kept contact to the adult care centre. Metabolic control was stable over the evaluation period and dried blood phenylalanine concentrations mostly remained within the therapeutic range (median 673.0 μmol/l, range 213.0–1381.1). Sociodemographic data, economical welfare and life satisfaction data were comparable to data from the general population. However, differences could be revealed when splitting the cohort according to time of diagnosis and to management during childhood. 83 % of the PKU adults were satisfied with the transition process and current adult care. 25 completed pregnancies were supervised; three newborns, born after unplanned pregnancy, showed characteristic symptoms of maternal PKU syndrome.

**Conclusions:**

Continuous care for adult PKU patients in a specialized outpatient clinic is successful, leading to good to satisfactory metabolic control and social outcomes. Uninterrupted good metabolic treatment throughout childhood and adolescence positively influences educational, professional and economic success in later life. Further effort in specialized paediatric and adult metabolic care is needed to prevent loss of follow-up and to support the recommended life-long treatment and/or care.

## Background

The need for consistent life-long care for chronically ill patients with special health care issues is increasingly recognized and requires further attention and development [1]. Especially the transition of adolescents from paediatric to adult health care is highly vulnerable [2–4]. They face new responsibilities for their own health care, calling for personal initiative and independence with respect to their disease management. In this situation, they are at risk to drop out of medical care [5, 6]. This is especially true for patients with inherited metabolic diseases, such as phenylketonuria (PKU; OMIM 261600) [7–9]. So far, the majority of adults with PKU are still treated in paediatric centres [8]; specialized adult centres are rare [8, 9]. Current guidelines recommend life-long treatment and medical attention to obtain the best possible neurologic outcome and to avoid comorbidities [10–12]. In addition, with an increasing number of PKU patients reaching reproductive age, the prevention of maternal PKU syndrome, a severe embryopathy affecting the unborn child of insufficiently controlled PKU mothers, is of increasing importance [13].

To overcome these challenges, an adult outpatient clinic and a coordinated transition process for patients with inherited errors of metabolism was established in 2005 in a cooperation of the paediatric outpatient clinic and the department of internal medicine at the University Hospital of Leipzig, Germany [9]. Since then, all patients 18 years and older with inherited metabolic diseases were transferred from paediatric to adult care. The adult metabolic care includes regular clinic visits and laboratory controls as well as dietary counselling and educational training.

The aim of this study was to evaluate the appropriateness of specialized adult metabolic care 10 years after its establishment. For this purpose, we retrospectively analysed medical records with respect to metabolic control, frequency of clinic visits, and adherence to treatment. Also, data on pregnancies and pregnancy outcome as well as sociodemographic data and educational status were recorded. In addition, a cross-sectional survey was performed regarding current therapy, life satisfaction and satisfaction with the transition process and adult care.

## Methods

### Patients

All PKU patients transferred from paediatric to adult care between 2005 and 2015 and followed in the adult outpatient clinic for inherited metabolic diseases, University of Leipzig, Germany were identified and included in the data analysis. Sociodemographic data as well as metabolic data and frequency of clinic visits were retrieved from medical records and analysed retrospectively. For better interpretation, all included patients were divided into three groups, according to the time of diagnosis and treatment regime: group A was diagnosed before implementation of newborn screening or beyond newborn period (age range 3 months to adulthood), group B was diagnosed by newborn screening (≤4 weeks of age) and treated early, with therapy interruption during childhood and adolescence for more than 4 years (start of interruption between 7–14 years of age, according to prior guidelines, effective in the German Democratic Republic [14]), and group C was diagnosed by newborn screening (≤4 weeks of age) and continuously treated throughout childhood and adolescence.

In addition, all completed pregnancies in PKU mothers over the evaluation period were recorded.

As an extension of the evaluation, a survey was performed in a sub-cohort. Inclusion in this survey was restricted to PKU patients with a minimum age of 18 years, confirmed diagnosis of PKU and therapy from the neonatal period (with or without interruption during childhood). Patients with other severe chronic diseases (i.e. metabolic diseases, epilepsy), concomitant oral medication except the phenylalanine (Phe) free amino acid mixture (AAM), or implementation of other diets except the PKU diet were excluded to avoid confounding effects. Also, PKU women currently pregnant or planning pregnancy were excluded from the survey for the same reason.

The study followed the principles of the guidelines in the World Medical Association Declaration of Helsinki of 1975, as revised in 2000 and the harmonised ICH-Guideline for Good Clinical Practice. It was approved by the University of Leipzig’s ethics committee (registration number 440-12-17122012) and registered at the International Clinical Trials Registry Platform (DRKS00004942). Patients who participated in the survey gave written informed consent.

### Metabolic control

To evaluate patients’ metabolic control, their annual individual average Phe concentrations in dried blood after transfer were calculated retrospectively. Every year, all patients with at least one contact with the metabolic centre (dried blood sample and/or clinic visit) were included in the final analysis. Analysis of Phe concentrations from dried blood spots was performed by liquid chromatography/tandem mass spectrometry (LC-MS/MS) as described [15].

### Survey

Between January 2013 and September 2015, all patients attending the outpatient clinic were screened for participation in the cross-sectional survey. Based on the questionnaire used in a prior transition study [9], a questionnaire was developed covering sociodemographic data (parenthood, educational achievement, employment, and net income), data on therapy management, dietary habits and metabolic control as well as satisfaction with transition and specialized adult care.

In addition, life satisfaction was assessed by “Questions on Life Satisfaction” (FLZm) [16], a standardised and validated questionnaire comprising the eight dimensions: friends, recreational activities, health, income/financial security, profession/work, domestic circumstances, family life/children, partnership/sexuality. In the evaluation process, the ratings for importance and satisfaction within the different dimensions are combined to receive information about weighted satisfaction, varying between -12 and +20 points. Global life satisfaction corresponds to the sum of the weighted satisfaction values with a maximum of 160 points.

### Statistical analysis

All statistical analyses were performed using SPSS for Windows 20 (SPSS Inc., Chicago, IL). Data are presented as median and range or as mean and standard deviation (SD), depending on data distribution. Mann-Whitney-*U*-test or *t*-test for independent samples were used to compare two groups. When more than two groups were compared, ANOVA was applied (with post-hoc Bonferroni test). Categorical data were analysed using the Chi square test. Significance was accepted for *P* < 0.05.

## Results

### Patients

Between October 2005 and March 2015, 96 adults with PKU (56 females/40 males; median age at time of analysis 32 years, range 18–62) were transferred from paediatric to specialized adult care.

The total number of patients with contact to the outpatient clinic slightly varied from year to year. In the past 3-year period, 81 % (44 females/32 males) of the transferred patients kept contact to the adult outpatient clinic. Contact was completely lost in 18 transferred patients. Seven of them informed us that they attended specialized care in a different centre due to vocational training or professional or personal reasons. No information was available for the remaining 11 patients despite regular effort to contact them.

### Metabolic control

The median of individual mean dried blood Phe concentrations of all transferred PKU patients did not differ over the 10-year period (673.0 μmol/l, range 213.0–1381.1) and dried blood Phe concentration was within the therapeutic range (according to the current recommendations for the German speaking countries [17]) most of the time. Phe concentrations were not significantly different between females and males. Median dried blood tyrosine concentrations (58.5 μmol/l, range 29.2–152.8) indicated an adequate supply. Dividing the cohort into the three groups (A, B, C) revealed some differences in metabolic control. Early diagnosed and continuously treated patients (group C) showed better metabolic control throughout the observation period than those with therapy interruption during childhood (group B). Interestingly, these differences were present (reaching statistical significance: *P* = 0.011 [ANOVA], driven by the difference of groups B and C: *P* = 0.016 [Bonferroni]) at the age of 6 years, i.e. even before treatment regimes diverged. Late diagnosis (group A) and therefore late introduction of the strict dietary treatment does not preclude good metabolic control (Table 2).

Since 2005, 25 completed pregnancies were supervised. Three newborns, born after unplanned pregnancy, showed characteristic symptoms of maternal PKU syndrome (Table 1). PKU patients with unplanned pregnancies exhibited significantly higher dried blood Phe concentrations than women with planned pregnancies (*P* < 0.001) and had significantly fewer contacts with the metabolic centre (visits and/or dried blood samples).

**Table 1:** Metabolic control of PKU females during pregnancy, comparing dried blood Phe concentrations in planned and unplanned pregnancies (Mann-Whitney *U*-test)

Metabolic control during pregnancy	planned *n* = 16	unplanned *n* = 9	P
Median (range) number of laboratory controls	36 (17–47)	17 (1–30)	<0.001
Median (range) dried blood Phe concentration μmol/l	181 (101–485)	476 (172–878)	<0.001
Offspring with clinical signs of maternal PKU syndrome	0	3	

### Sociodemographic data

The majority of the adult PKU patients lived in the German federal state of Saxony, at distances up to 400 km from the metabolic centre. Sociodemographic data of the PKU cohort are shown in Table 2 in comparison to the annual statistics of Saxony for 2014 [18, 19]. While a slightly higher proportion of PKU patients quit school without formal graduation, educational attainment was similar to that of the general population. Income tended to be lower in PKU patients. Subgroup analysis showed that the majority of patients diagnosed late (group A) attended schools for special education and only one of these patients completed an apprenticeship. Patients without therapy interruption (group C) reached higher graduation levels, and even a slightly higher proportion achieved university graduation, as compared to the general population; presumably due to the sample size, chi square test was non-significant. PKU did not discourage patients from establishing a family, independent of prior therapy management. No parenthood was documented in the group of late diagnosed PKU so far.

**Table 2:** Sociodemographic data and metabolic control of the investigated adult PKU cohort as well as for subgroups (group A = late diagnosed patients; group B = early diagnosed + therapy interruption ≥ 4 years during childhood/adolescence; group C = early diagnosed and continuously treated) compared to the reference population of German federal state of Saxony [18, 19]. Graduations: modern certificate (i.e. at the age of 15 years; after 9 years of school), secondary school certificate (i.e. at the age of 16 years; after 10 years of school), high school diploma (i.e. at the age of 18–19 years; after 12–13 years of school). The number of PKU patients per characteristic varies, as not all data of all patients were available

	PKU patients total (*n* = 96)	Group A (n^1^ = 8)	Group B (n^1^ = 29)	Group C (n^1^ = 59)	Population of Saxony 2014
Current median age (range) in years	32 (18–62)	32 (19–62)	39 (34–46)	30 (18–44)	n.a.
Number currently on/off diet (n)	76/20	6/2	21/8	49/10	n.a.
Graduation	n^2^ = 90	n^2^ = 7	n^2^ = 28	n^2^ = 55	
no graduation/special education [%]	14.5^a^	85.7	10.7	3.6	8.7
secondary modern school certificate [%]	18.9	14.3	28.6	18.2	9.9
secondary school certificate [%]	43.3	0.0	50.0	45.5	52.0
high school diploma [%]	23.3	0.0	10.7	32.7	29.4
Educational attainment	n^2^ = 85	n^2^ = 7	n^2^ = 28	n^2^ = 51	
Apprenticeship [%]	77.6	14.3	89.3	76.5	77.2
University degree [%]	12.9	0.0	7.1	17.6	14.5
Without [%]	10.6^a^	85.7	3.6	5.9^c^	8.3
Net income (per month)	n^2^ = 24^b^	n.a.	n^2^ = 8^b^	n^2^ = 16^b^	
<700€	20.8		12.5	25.0	13.0
700€–1500€	50.0		62.5	43.8	53.5
>1500€	29.2		25.0	31.2	33.5
Parenthood [%]	n^2^ = 78	n^2^=80.0	n^2^ = 23	n^2^ = 48	41.0
	41.0		56.5	39.6	
Metabolic control					
Median (range) dried blood Phe concentration during the 6^th^ year of life [μmol/l]	n^2^ = 74	n^2^ = 3	n^2^ = 23	n^2^ = 48	n.a.
	307.3 (92.1–1246.6)	203.9 (192.9–289.7)	447.0 (107.9–1246.6)	263.6 (92.1–901.9)	
Median (range) dried blood Phe concentration during the 18^th^ year of life [μmol/l]	n^2^ = 71	n^2^ = 4	n^2^ = 19	n^2^ = 48	n.a.
	587.4 (52.5–1454.6)	650.2 (434.8–1247.0)	622.3 (169.7–1454.6)	562.6 (52.5–1186.6)	
Current median (range) dried blood Phe concentration (last three values) [μmol/l]	n^2^ = 96	n^2^ = 8	n^2^ = 29	n^2^ = 59	n.a.
	658.7 (109.1–1458.5)	617.9 (323.4–1209.7)	789.0 (109.1–1458.5)	596.2 (168.1–1365.2)	

n^1^: all retrospectively analysed patients

n^2^: patients with available data

n.a. not applicable

^a^includes late diagnosed PKU patients with special school education and patients still in education

^b^data available only for the participants of the survey (*n* = 30)

^c^in total three patients, two of them are still trainees

### Survey

During the survey period (January 2013 until September 2015), 67 patients attended the clinic at least once. Of these, 46 PKU patients fulfilled the inclusion criteria and were asked to participate, 21 were excluded due to late diagnosis (*n* = 5), comorbidities (*n* = 9) or pregnancy and pregnancy planning (*n* = 7). A total of 30 patients (17 females/13 males; median age 32 years, range 18–43) responded to the survey.

Results of the survey are presented in Table 3. Patients currently on diet showed significantly lower mean dried blood Phe concentrations (*n* = 22; 637 ± 212 μmol/l, range 282–1072) compared to patients off diet (*n* = 8, 842 ± 174 μmol/l, range 530–1118, *P* = 0.015, dried blood Phe concentrations 1 year prior evaluation). Median tyrosine concentrations were significantly higher in patients on diet compared to patients off diet (66.9 μmol/l, range 37.0–162.5 vs 42.1 μmol/l, range 32.8–67.4, *P* = 0.006). Interestingly, all but two patients off diet belonged to the group with therapy interruption of at least 4 years during childhood.

**Table 3:** Therapy management and satisfaction (single centre survey; *n* = 30; 17 females/13 males). A Clinic visits, laboratory controls and dietary treatment. B Transition satisfaction and coping with treatment

A: Clinic visits, laboratory controls and dietary treatment		
Frequency of laboratory controls/dried blood samples	n	%
Monthly	13	43.3
Every two month	5	16.7
Quarterly	5	16.7
Biannually	3	10.0
Annually	2	6.7
No answer	2	6.7
Frequency of clinic visits		
Quarterly	11	36.7
Three times a year	2	6.7
Biannually	12	40.0
Annually	4	13.3
No visits	1	3.3
Dietary treatment		
Phe/protein restricted diet	22	73.3
*Exact calculating Phe content*	4	12.3
*Estimation of Phe content*	16	53.3
*No strict diet regime/vegetarian diet*	2	6.6
Amino acid mixture (AAM)	24	80.0
BH4	0	0
Discontinuation of the PKU treatment		
Never	14	46.7
Once	7	23.3
Several times	5	16.7
No answer	4	13.3
Reasons for discontinuation of the PKU treatment (*n* = 12)		
Stop of dietary treatment in adolescence according to former procedures	3	25.0
Weariness to follow the diet/Wish for independence from treatment	5	41.7
Good metabolic control following a vegetarian diet	3	25.0
No answer	1	8.3
Restart of the PKU treatment	10	83.3
Reasons for restart of the PKU treatment (*n* = 10)		
Doctors recommendation	2	20.0
More capability/long-term prevention	2	20.0
Wish to become pregnant/pregnancy	4	40.0
No answer	2	20.0
B: Transition satisfaction and coping with treatment.		
Satisfaction with the actual individual metabolic control	n	%
Very satisfied	10	33.3
Satisfied	14	46.7
Less satisfied	5	16.7
Not satisfied	0	0
No answer	1	3.3
Personal importance of good metabolic control		
Very important	12	40.0
Important	14	46.7
Less important	3	10.0
Not important	0	0
No answer	1	3.3
Current coping with dietary therapy		
Very well	10	33.3
Well	13	43.3
Moderately	2	6.7
Badly	1	3.3
No answer	4	12.3
Coping with dietary treatment in adulthood in comparison to childhood		
Easier	13	43.3
Indifferent	8	26.7
More difficult	5	16.7
No answer	4	12.3
Satisfaction with transition process and adult care		
Very satisfied	10	33.3
Satisfied	15	50.0
Less satisfied	0	0
Not satisfied	3	10.0
No answer	2	6.7

Twenty-seven participants (16 females/11 males) answered the “Questions on Life Satisfaction”, three declined. The respondents’ mean score was 66.7 ± 31.7 points, compared to an average value of the German population of 62.7 ± 37.1 points (age group 18 to 45 years) [16]. The higher score in PKU females was not statistically different from their male counterparts (70.2 ± 36.5 vs. 61.6 ± 23.7 points; *P* = 0.468). Patients on diet exhibited higher scores of life satisfaction compared to patients off diet (72.9 ± 33.9 vs. 52.0 ± 20.5 points, *P* = 0.062).

## Discussion

Transition from paediatric to adult health care is a particularly vulnerable period for patients with inherited metabolic diseases [20]. Data revealed so far show the need for a special education in inherited metabolic diseases for adult health care providers, new guidelines for adult patients, and an improvement of the structures for transition [7–9, 11, 21]. Most PKU adults are cared for in paediatric centres [8] and there are only few specialized adult outpatient clinics. Therefore, only limited data about PKU adult care are available. In 2005, a transition program and outpatient clinic for adults with inherited metabolic diseases was established in Leipzig [9]. Now, a retrospective data analysis and cross-sectional survey evaluating the first 10 years of specialized adult PKU care was performed.

Analysing clinical practice in a specialized adult outpatient clinic for inherited metabolic diseases over a 10-year period, 81 % of the transferred PKU patients still kept contact to the adult care centre in the last 3 years. Metabolic control was stable over the evaluation period and dried blood Phe concentrations mostly remained within the therapeutic range [17]. Overall sociodemographic, economic and life satisfaction data were comparable to the reference population and 83 % of the PKU adults were satisfied with the transition process and adult care.

Frequency of contact to the metabolic centre varied widely. Some patients did not attend the clinic for some time only to come back for regular care after a while. Looking at 3-year periods, about 80 % of all transferred patients had at least one contact. Due to vocational training or professional and personal reasons, some patients moved to other metabolic centres. Less than 12 % appear to be lost to the specialized care. These data indicate that a successful, continuous follow-up of adult PKU patients is achievable. With a median age of 32 years, our cohort extends previously reported experience from an international survey of adults with PKU, reporting outcomes at a median age below 29 years [8].

During the 10-year follow-up, all supervised PKU patients showed good metabolic control with individual mean Phe concentrations within the therapeutic range according to the current guidelines for the German speaking countries [17]. No significant difference in metabolic control during adulthood could be detected with respect to age at diagnosis or therapeutic strategy during childhood/adolescence. However, as expected, the analysis of the survey yielded significantly higher Phe concentrations in patients off diet compared to those on diet. Patients with optimal metabolic control were also those who attended the clinic more often (data not shown). This indicates that realisation of recommended therapeutic targets, which were shown to improve outcome [22, 23], requires constant patient education. In light of the recently updated US guidelines and the expected European guidelines, which both advocate even stricter metabolic targets, this issue becomes even more important [23]. This stricter recommendation might improve patients’ outcome, but will also carry an even greater burden for the patients to successfully adhere to treatment. Former studies showed therapy adherence declined especially during adolescence and adulthood [9, 24]. In the present survey, half of the adult patients interrupted therapy at least once over an extended period (>4 years). Reasons were former guidelines in the eastern part of Germany, no longer in place [14], but also the typical difficulties in treatment of adolescents and young adults with chronic diseases. Nevertheless, the majority of these patients restarted therapy after picking up regular consultations in the specialised metabolic centre.

Especially the supervision of PKU females in childbearing age represents a great challenge [13]. For this group of patients, strict metabolic control with Phe concentrations between 120 and 360 μmol/l is indispensable before and during pregnancy in order to prevent maternal PKU syndrome [25]. The majority of the evaluated pregnancies in this cohort were planned, well supervised and, therefore, under good metabolic control, in accordance to the current recommendation [25]. One-third of the pregnancies, however, were unplanned and, therefore, occurred under unfavourable metabolic conditions: in unplanned pregnancies significantly higher Phe concentrations were recorded compared to planned pregnancies. This again underscores the importance of specialized adult care in PKU adults. All three children with maternal PKU syndrome were born after unplanned pregnancy with insufficient metabolic control [26]. Unfortunately, the mothers refused to follow any of the recommendations, despite every effort by the metabolic team. Importantly, these documented cases of maternal PKU syndrome might not be representative of the entire scope of maternal PKU syndrome even in our cohort. Since clinically normal infants from PKU mothers are not routinely followed up so far, the incidence of mild forms may be underestimated [27].

Successful transition requires an experienced multidisciplinary team of paediatric and adult health care providers. The majority of the included patients were satisfied with their transition process and care during the last 10 years and cope well with their therapy. For most, adherence to therapy is easier as adults than during childhood. However, especially female patients expressed the wish for an even more intensive care, referring to dietary counselling, real-time information about Phe values and a closer contact for following therapy adaptions (data not shown).

Most of the survey participants still followed a Phe restricted diet and took AAM, albeit not as strictly as during childhood. Moreover, the majority reported good metabolic control to be personally important and were satisfied with their current metabolic control.

Regarding life satisfaction, all patients showed a good general life satisfaction with a mean score above the score of a German reference sample [16]. This is in accordance with former findings in patients with PKU [9, 28]. One might question the relevance of these data as the investigated group is rather small. However, in order to exclude potential confounding factors, strict inclusion criteria were implemented. Life satisfaction of patients on diet was higher with a score difference of 20 points compared to patients off diet. Interestingly, patients on diet scored higher than the general population mean, while those off diet scored lower than the general population mean by the same degree. Due to high variance of scores in patients and the general population, these differences missed significance. This important aspect of therapy continuation is in accordance with a former study reporting that patients’ quality of life improved after reintroduction of a Phe-restricted diet [29]. The resumption of the Phe-restricted diet, however, often represents a great challenge for the patients as they show low therapy adherence or terminate the diet again after a short while [29]. Again, this supports the recommendations for continuous therapy and adult specialised care.

Social outcome acts as an important indicator for the effective life-long treatment of inherited metabolic diseases. In a previous study, we reported some minor differences regarding school graduation compared to the reference population [9]. This is in accordance with similar studies by other groups [30–33]. Despite important improvements in therapeutic management over the past decades, differences of sociodemographic development remain between PKU patients and the control population. This depends not only on time of diagnosis, but also on continuity of consistent treatment throughout childhood, adolescence and the transition period. As expected, patients diagnosed late did not visit regular school, and consequently did not reach a general educational degree. Early diagnosed patients who interrupted their treatment during childhood and adolescence for several years performed markedly poorer with respect to school graduation and educational attainment. In contrast, early diagnosed and continuously treated patients showed no differences in socioeconomic data compared to the healthy population. In some fields they even perform better, as shown by the very low rate of these young PKU adults leaving school without graduation.

The distribution of net income was comparable between early diagnosed PKU patients and the control cohort independent from therapy interruption. It improved since 2008, correcting for inflation and the general economic situation in the state of Saxony [9, 18, 19]. Fortunately, and in contrast to former studies reporting rare parenthood in adult PKU patients [9, 30], the percentage of adults with PKU having children was comparable to the reference population. PKU seems no longer to discourage patients from establishing a family.

The cohort size of the survey was limited by strict inclusion criteria. The lower percentage of patients on diet than in our prior study (73 % vs. 92 %) [9] is influenced by exclusion of women with planned or current pregnancies. As always, a selection bias by inclusion of motivated patients must be considered, especially for the survey data.

The obvious limitation of a single-centre study can currently not be overcome since adult PKU centres are only being established, so that comparable longitudinal data are not available from other sites. Analysis of larger study groups, once available, may add important and interesting aspects.

## Conclusion

Continuous care for adult PKU patients in a specialized outpatient clinic is successful, leading to good to satisfactory metabolic control and social outcomes. Uninterrupted consistent metabolic treatment throughout childhood and adolescence positively influences educational, professional and economic success in later life. Despite enormous efforts, prevention of maternal PKU syndrome remains a challenge. Further effort in specialized paediatric and adult metabolic care is needed to prevent loss of follow-up and to support the recommended life-long treatment and/or care.

### Ethics statement

Patients participated in the survey after informed consent.

## Abbreviations

AAM: amino acid mixture
Phe: phenylalanine
PKU: phenylketonuria
